# Correlation Analysis and Spiking Validation of Flavor Compounds Modulating Bitterness and Astringency in Jasmine Tea

**DOI:** 10.3390/foods15173047

**Published:** 2026-08-28

**Authors:** Yi-Jie Liu, Jia-Ying Chen, Mei-Juan Hu, Jian-Hui Ye, Wen-Wen Luo

**Affiliations:** 1 Tea Research Institute, Zhejiang University, Hangzhou 310013, China; 22416177@zju.edu.cn (Y.-J.L.); 22316222@zju.edu.cn (J.-Y.C.); 2 Zhejiang Huamingyuan Tea Co., Ltd., Jinhua 321000, China; 3 Jinhua Department of Economic Specialty Technology Promotion, 828 Shuanglong South Road, Jinhua 321000, China

**Keywords:** jasmine tea, WGCNA, bitterness and astringency modulation, spiking experiment

## Abstract

To elucidate the chemical basis underlying bitterness and astringency modulation in jasmine tea, four green tea bases and their corresponding jasmine teas were analyzed using weighted gene co-expression network analysis (WGCNA) combined with spiking validation experiments. WGCNA revealed that catechin, gallocatechin gallate, aspartic acid (Asp), glutamic acid (Glu), arginine (Arg), and alanine (Ala) were positively associated with sweetness and umami but negatively associated with bitterness and astringency. Spiking experiments showed that taste modulation was both concentration- and matrix-dependent. Glu and Asp effectively reduced bitterness at suitable concentrations, whereas the effects of theanine and Arg varied among tea matrices. Bitterness and astringency of jasmine tea infusion are modulated by the combined effects of the chemical matrix of tea base, providing a theoretical basis for tea-base selection and jasmine tea flavor optimization.

## 1. Introduction

Jasmine tea is a reprocessed tea produced by scenting a tea base with fresh jasmine flowers and is one of the most representative categories of traditional Chinese scented tea [1,2]. Jasmine flowers are particularly suitable for tea scenting because they are rich in aromatic compounds, including terpenes and indoles, which contribute to the characteristic floral aroma of jasmine tea [3,4]. However, the quality of jasmine tea is determined not only by its characteristic aroma but also by its taste, with the tea base serving as the primary source and foundation of its taste characteristics. Therefore, the balance among aroma, taste, and mouthfeel is a critical dimension of tea quality evaluation [5,6,7]. The bitterness and astringency of tea infusions are determined not only by the concentrations of caffeine, catechins, polyphenols, amino acids, and other taste-active compounds [8] but also by their complex interactions, which can modify taste perception and ultimately shape the overall flavor profile of the tea infusion [9]. The perceived intensities of these taste attributes depend on the molecular structures and concentrations of the contributing compounds, as well as on their interactions with other constituents in the complex chemical matrix of the tea infusion [10,11,12]. For jasmine green tea, the green tea base not only serves as the primary source of taste-active compounds but also provides a complex chemical matrix in which these compounds interact with constituents introduced during scenting [13], thereby strongly influencing the final taste profile of the scented tea.

Bitterness and astringency are key attributes determining the taste quality of jasmine tea, the perceived intensities of which are potentially modifiable [14]. Previous studies have shown that perceived bitterness and astringency can be modulated through multiple, partly distinct mechanisms, including inhibition of bitter-taste receptor signaling, physicochemical interactions among taste-active compounds and oral constituents, taste–taste mixture interactions, and cross-modal odor–taste effects [15,16,17]. For example, umami peptides can attenuate bitter compound-induced hT2R16-mediated Ca^2+^ signaling [18]. Polyphenols can interact with proteins and certain amino acids via non-covalent interactions, primarily hydrophobic interactions and hydrogen bonding. These interactions may reduce the availability of unbound polyphenols for oral perception and modulate their sensory expression, thereby altering the perceived bitterness and astringency of the system [15,19,20,21]. Glutamic acid has also been reported to suppress epigallocatechin gallate (EGCG) bitterness more effectively than theanine [16], whereas sucrose can reduce bitterness through mixture suppression effects [22,23]. These findings indicate that the effects of taste-modulating compounds depend on both their concentrations and the chemical matrix in which they occur.

Although bitterness and astringency in unscented tea infusions have been examined, regarding chemical composition, processing-induced changes, and taste interactions [8,24], the key compounds regulating these attributes in jasmine teas produced from different tea bases remain unclear. Differences in catechins, caffeine, flavonol glycosides, and free amino acids among green tea bases may influence not only the inherent bitterness and astringency of tea infusions but also the masking and modulatory effects of other taste-active compounds [21,25]. In this study, jasmine teas were prepared from four green tea bases under two scenting temperatures (30 °C and 35 °C). The compositions of major flavor compounds in different tea bases and jasmine tea samples were analyzed, and six sensory attributes, including smoothness, sweetness, umami, astringency, thickness, and bitterness, were assessed by quantitative descriptive analysis (QDA). Weighted correlation network analysis (WGCNA) was used for screening candidate compounds associated with six sensory attributes. Spiking experiments were performed to validate the sensory effects of selected candidate compounds within the tea infusion matrix. The study aimed to elucidate the chemical basis of bitterness and astringency modulation in jasmine tea and provide a theoretical basis for tea base selection and flavor optimization.

## 2. Materials and Methods

### 2.1. Materials and Chemicals

Tencha was supplied by Lanxi Chishan Lake Green Farm Co., Ltd. (Lanxi, China). The curved-strip Maofeng tea was supplied by Wuyi Tangji High Mountain Tea Industry Co., Ltd. (Wuyi, China). The orchid-shaped Maofeng tea was obtained from Lanxi Xiacen Maofeng Tea Factory (Lanxi, China), while the flat-shaped green tea was supplied by Wuyi Shushui Tea Industry Co., Ltd. (Wuyi, China). Jasmine teas prepared from the four green tea bases were manufactured by Zhejiang Wuzhou Tea Co., Ltd. (Jinhua, China), according to the Chinese national standard GB/T 34779-2017 [26] and the company’s standard processing procedures.

Individual catechins (all ≥98%), including epigallocatechin gallate (EGCG), epigallocatechin (EGC), epicatechin gallate (ECG), epicatechin (EC), gallocatechin gallate (GCG), gallocatechin (GC), catechin gallate (CG) and catechin (C), as well as caffeine (≥98%) were purchased from Sigma-Aldrich (Shanghai, China). Flavonol glycosides, including myricetin-3-*O*-galactosyl-rhamnosyl-glucoside (M-gal-rha-glu), myricetin 3-*O*-galactoside (M-gal), myricetin 3-*O*-glucoside (M-glu), quercetin-3-*O*-galactosyl-rhamnosyl-glucoside (Q-gal-rha-glu), quercetin-3-*O*-glucosyl-rhamnosyl-glucoside (Q-glu-rha-glu), quercetin-3-*O*-rhamnosyl-rhamnosyl-glucoside (Q-glu-rha-rha), quercetin 3-*O*-galactoside (Q-gal), quercetin 3-*O*-glucoside (Q-glu), kaempferol-3-*O*-glucosyl-rhamnosyl-glucoside (K-glu-rha-glu), kaempferol-3-*O*-galactoside (K-gal), kaempferol-3-*O*-rhamnosyl-glucoside (K-glu-rha), kaempferol 3-*O*-glucoside (K-glu) were prepared in our laboratory according to previously published methods [27]. Amino acid standards, including aspartic acid (Asp), L-glutamic acid (Glu), L-asparagine (Asn), L-serine (Ser), L-glutamine (Gln), L-histidine (His), glycine (Gly), threonine (Thr), arginine (Arg), L-alanine (Ala), γ-aminobutyric acid (GABA), L-theanine (Thea), L-tyrosine (Tyr), L-valine (Val), methionine (Met), L-norvaline (Nva), L-tryptophan (Trp), L-phenylalanine (Phe), L-isoleucine (Ile), L-leucine (Leu), and L-lysine (Lys), were purchased from Aladdin Reagent Co., Ltd. (Shanghai, China). HPLC-grade acetonitrile and methanol were purchased from Merck KGaA (Darmstadt, Germany). Ultrapure water was prepared using an EASY Pure II UV water purification system (Barnstead International, Dubuque, IA, USA).

### 2.2. Preparation of Jasmine Teas from Different Tea Bases

Jasmine tea samples were prepared from each of the four tea bases through three consecutive scenting rounds at 30 or 35 °C. For each tea base, the jasmine tea samples produced at 30 and 35 °C were designated S30 and S35, respectively. Each round was conducted for 16 h, using a green tea-to-fresh jasmine flower mass ratio of 10:7. Apart from scenting temperature, all processing parameters were kept consistent among the tea bases and treatments. After the third round, the jasmine flowers were removed by sieving, and the scented tea leaves were dried. The dried jasmine tea samples were stored at −20 °C prior to analysis.

### 2.3. Tea Infusion Preparation

Tea infusions were prepared according to the Chinese national standard for sensory evaluation of tea (GB/T 23776-2018) [28]. Briefly, 3 g of each tea sample was infused with 150 mL of boiling water for 4 min.

### 2.4. Sensory Evaluation

QDA was conducted to evaluate the sensory attributes of each sample, focusing on smoothness, sweetness, umami, astringency, thickness, and bitterness. Sensory evaluation was performed by a trained panel of six tea tasters (Occupational Code: 6-02-06-11). Before the formal evaluation, panelists underwent multiple training sessions to familiarize themselves with the attribute definitions, reference standards, and scoring procedures. To improve sensory reliability, standard solutions with different concentration levels were prepared for calibration of taste intensities, enabling panelists to establish consistent sensory references for sweetness, umami, bitterness, astringency, thickness, and smoothness. Additional calibration sessions were conducted until the panel reached stable evaluations and acceptable agreement among assessors.

Sensory evaluations were performed in a sensory evaluation room under the conditions of 25 ± 2 °C, relative humidity 50–60%, and neutral lighting. For tea infusion evaluation, 30 mL of each tea infusion was served in odorless cups labeled with randomly assigned three-digit codes. Samples were presented in a randomized order for each assessor, and the evaluation was conducted under blinded conditions. Water was provided for palate cleansing. Each taste attribute was rated by the panelists on an 11-point intensity scale, with 0 indicating absence and 10 indicating extremely high intensity. Selected samples were evaluated repeatedly in independent sessions to ensure measurement repeatability, and the final sensory scores were calculated as the average values across all assessors.

For the spiking experiments, the spiked tea infusion samples were evaluated using the same blinded, randomized, and calibrated sensory procedure. The sensory scores obtained from the panel were analyzed to determine the potential contribution of individual compounds to the overall flavor characteristics of tea infusions. The mean score for each attribute was calculated and used for subsequent statistical analysis and radar-chart visualization.

### 2.5. Determination of Flavonoids and Caffeine

The tea infusion prepared as described above was centrifuged at 12,000 rpm and 4 °C for 20 min, and the supernatant was collected for analysis. The concentrations of catechins, flavonol glycosides, and caffeine were determined by an Ultra-performance liquid chromatography coupled with diode-array detection and tandem mass spectrometry (UHPLC-DAD-MS/MS) system, according to our previously published method [24]. Flavonol glycosides, catechins, and caffeine were quantified using their respective authentic standards and an external standard calibration method. Detection wavelengths were set at 360 nm for flavonol glycosides and 280 nm for catechins and caffeine.

### 2.6. Determination of Amino Acids

Amino acids were determined according to a previously reported method [27], with minor modifications. Briefly, the sample solution was mixed sequentially with 1 mL of borate buffer (0.4 mol/L, pH 10.2), 200 μL of *o*-phthalaldehyde (OPA) derivatization reagent, 780 μL of ultrapure water, and 10 μL of norvaline as the internal standard. The OPA reagent was prepared by dissolving 0.01 g of OPA in a mixture of 1 mL of acetonitrile, 9 mL of borate buffer (0.4 mol/L), and 100 μL of 3-mercaptopropionic acid. The mixture was filtered through a 0.22 μm membrane, and then submitted to HPLC analysis using following conditions: ZORBAX Eclipse AAA column (4.6 × 150 mm, 3.5 μm), column temperature 40 °C, injection volume 10 μL, mobile phase A: 40 mmol/L sodium hydrogen phosphate buffer (pH 7.8), mobile phase B: acetonitrile/methanol/water (45:45:10, *v*/*v*/*v*; solvent B), flow rate 1.0 mL/min. The gradient elution program was as follows: 0–35 min, 5–60% B; 35–40 min, 60–100% B; 40–45 min, 100% B; 45–50 min, 5% B for column re-equilibration. Amino acids were detected by fluorescence at excitation and emission wavelengths of 340 and 450 nm, respectively, and quantified using the external standard method with the corresponding authentic standards.

### 2.7. Correlation of Flavor Components and Taste Attributes via WGCNA

The concentrations of major flavor compounds and the corresponding sensory scores of the 36 tea samples, including the four tea bases and their respective jasmine teas, were integrated to evaluate the associations between chemical constituents and sensory attributes. WGCNA was performed to construct the coexpression network of flavor compounds using the WGCNA package in R (version 4.2.2) according to the reported method [29]. Pairwise Pearson correlations among flavor compounds were used to generate the adjacency matrix, which was converted into a weighted adjacency matrix using a soft-thresholding power (β) of 14. The topological overlap matrix (TOM)-based dissimilarity was then used for hierarchical clustering, and modules were identified using the DynamicTreeCut algorithm. Module eigengenes were correlated with sensory scores.

### 2.8. Spiking Experiment

Jasmine tea samples prepared from strip-shaped Maofeng and Tencha through three scenting rounds at 35 °C were used for the spiking experiments. Each sample (3.0 g) was infused with 150 mL of boiling water for 4 min and then filtered. For each treatment, 28.5 mL of tea infusion was mixed with 1.5 mL of the corresponding standard solution. The compounds were added to the tea infusions at the following final concentrations: 0.01, 0.02, 0.03, and 0.04 mg/mL for Glu; 0.10, 0.20, 0.30, 0.40, and 0.50 mg/mL for Thea; 0.03, 0.06, 0.09, 0.12, and 0.15 mg/mL for Asp; 0.01, 0.03, 0.06, 0.09, and 0.12 mg/mL for Arg. The spiked tea infusions were evaluated by QDA under blinded conditions.

### 2.9. Statistics

All the experiments were conducted in triplicate, and the results were expressed as mean ± standard deviation (SD). Statistical analyses were conducted using IBM SPSS Statistics version 27.0 (SPSS Inc., Chicago, IL, USA). Normality and homogeneity of variance were checked before one-way ANOVA. For each tea base, the three samples subjected to different treatments were compared pairwise using Tukey’s HSD test following ANOVA. Significance was set at *p* < 0.05.

## 3. Results and Discussion

### 3.1. Correlation of Flavor Compounds and Taste Traits via WGCNA

Table 1 shows the concentrations of major flavor compounds in tea infusions prepared from different tea samples. Compared with their corresponding tea bases, the jasmine tea infusions showed compound-specific changes in chemical composition after scenting. The contents of total catechins (TC) and caffeine remained stable across the four tea types, suggesting that these compounds were hardly affected by scenting. The concentration of total flavonol glycosides (TFG) significantly increased after scenting, from 25.78–35.93 μg/mL in tea bases to 27.90–39.12 μg/mL in the corresponding jasmine teas. This increase was particularly pronounced in tencha and flat-shaped tea, which was mainly attributed to the elevated level of Q-glu-rha-rha (Table 1).

By contrast, total amino acids (TAA) content decreased greatly after scenting, from 621.17–904.26 μg/mL in the tea bases to 454.09–725.78 μg/mL in the scented teas. The reduction in TAA content was mainly attributed to decreases in theanine, aspartic acid, and glutamic acid levels. Amino acid degradation was generally more pronounced at 35 °C than at 30 °C, particularly in orchid-shaped Maofeng and Tencha.

**Figure 1:**
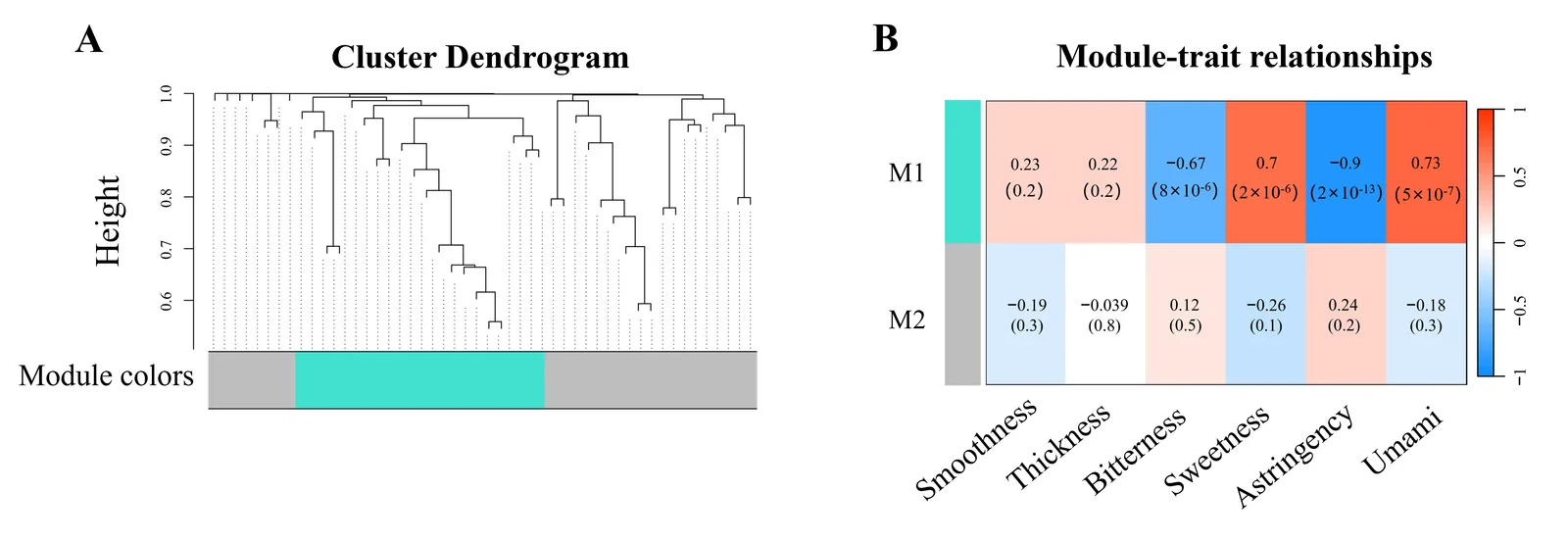
Association of chemical compounds with sensory traits via WGCNA. (**A**) Hierarchical clustering dendrogram and (**B**) Module-trait relationships.

WGCNA was used to explore the associations between flavor compounds and sensory traits. As shown in Figure 1, two modules (M1 and M2) were identified by hierarchical clustering. Module–trait relationship analysis revealed that the M1 module exhibited strong associations with multiple sensory quality traits. Specifically, M1 showed a significant negative correlation with bitterness (r = −0.67, *p* < 0.05) and astringency (r = −0.90, *p* < 0.05), while displaying significant positive correlations with sweetness (r = 0.70, *p* < 0.05) and umami (r = 0.73, *p* < 0.05). In contrast, the M2 module showed no significant correlations with any evaluated traits (*p* > 0.05). These results indicate that the M1 module represents a key co-expression module potentially associated with the modulation of tea sensory quality, particularly taste-related characteristics. Based on the thresholds of GS > 0.2 and MM > 0.8 [30], C, GCG, Asp, Glu, Arg, and Ala were identified as potential taste-related compounds, exhibiting positive correlations with sweetness and umami intensities and negative correlations with bitterness and astringency intensities. These statistical associations suggest their possible roles in shaping tea taste characteristics, although their sensory contributions require further confirmation.

The positive associations of Asp and Glu with umami are consistent with previous reports showing that free amino acids, particularly Asp and Glu, are important contributors to the umami quality of tea infusions. Ala may further contribute to a sweet or mellow taste impression, thereby enhancing the overall sweetness–umami profile [31]. These amino acids may reduce bitterness and astringency through interactions with bitter compounds like catechins [16]. Although Arg is generally considered as a bitter amino acid, its concentration in the tea infusions was far below the reported bitterness threshold of 0.21 mg/mL, suggesting that it likely makes a negligible direct contribution to bitterness perception [32]. Nevertheless, Arg may indirectly influence sensory quality by modulating bitter taste perception through the attenuation of bitter receptor-mediated Ca^2+^ signaling [32], and by serving as a precursor for aroma-active compounds generated during Maillard reactions [33]. Therefore, spiking experiments are warranted to further elucidate the contribution of Arg to tea flavor. Regarding catechin compounds, the negative associations of C and GCG with bitterness and astringency are more likely to reflect the overall sensory profile of the tea infusion matrix rather than the intrinsic taste properties of these individual catechins. It has been acknowledged that fresh tea leaves contained substantially lower levels of C and GCG than the processed or dried tea samples, since C and GCG can be formed through thermally induced epimerization of EC and EGCG during tea processing [34,35]. Since EC and EGCG generally show stronger astringency than their corresponding epimers C and GCG [36]. Thermally induced epimerization of EC and EGCG to C and GCG may improve the taste profile of processed green tea. This partly explains the negative associations of C and GCG with bitterness and astringency revealed by WGCNA.

### 3.2. Validation of the Taste-Modulating Effects of Glu

Based on the WGCNA results, Glu, Thea, Asp, and Arg were selected for spiking experiments to evaluate their effects on bitterness and astringency of tea infusion. Ala was excluded from further validation because its concentration in tea infusions (7.93–24.01 μg/mL) was substantially lower than its reported sweetness threshold (1.44 mg/mL), suggesting that it was unlikely to directly contribute to sweetness perception under the tested conditions. In addition, Ala accounted for less than 5% of the total amino acid content (TAA) [37]. Maofeng and Tencha were selected as representative tea matrices due to their distinct yet typical chemical profiles, particularly in terms of flavonoid and free amino acid compositions, which are closely associated with differences in bitterness and astringency.

**Figure 2:**
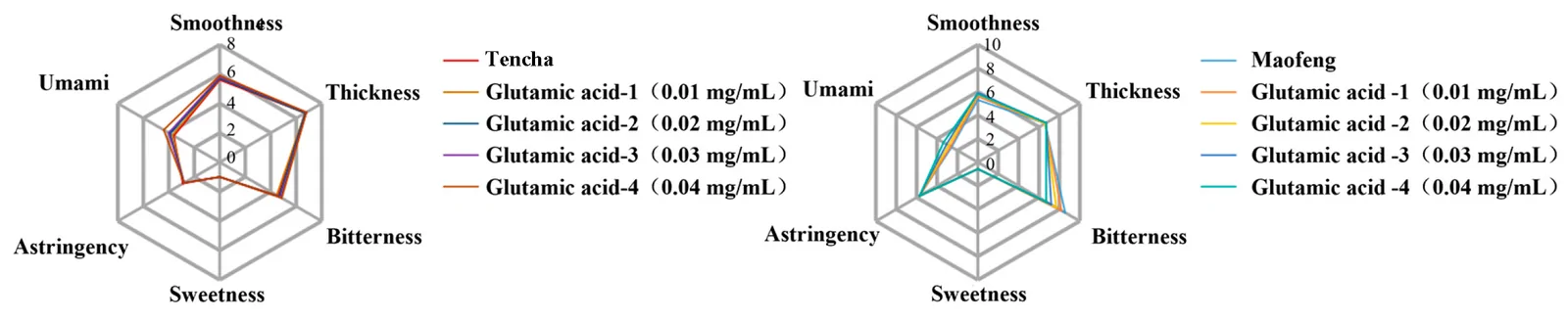
The effect of glutamic acid addition on the infusion taste of Tencha and Maofeng.

Figure 2 shows Glu exhibited matrix-dependent effects on bitterness. In Maofeng infusion, supplementation with 0–0.04 mg/mL Glu progressively reduced the bitterness score from 8.6 (control) to 6.8. By contrast, no bitterness-suppressing effect was observed in Tencha infusion, with the score slightly increasing from 4.6 to 4.9. Glu has a low umami threshold of approximately 0.01 mg/mL [38], indicating that small changes in its concentration may markedly affect taste perception. The different performances of these two tea infusions may be related to their chemical matrices. Glu has been reported to suppress the bitterness of EGCG more effectively when EGCG is present at 0.17 mg/mL, whereas its inhibitory effect is weaker at lower EGCG concentrations [16]. Based on calculation, the EGCG concentration in the spiked Maofeng infusion was higher than 0.17 mg/mL, whereas that in the spiked Tencha infusion was slightly below 0.17 mg/mL. This difference may partly explain why the bitterness score of the Maofeng infusion decreased markedly after the addition of Glu. The higher level of gallated catechins and lower level of amino acids in Maofeng may further enhance its responsiveness to Glu supplementation.

### 3.3. Validation of the Taste-Modulating Effects of Thea

**Figure 3:**
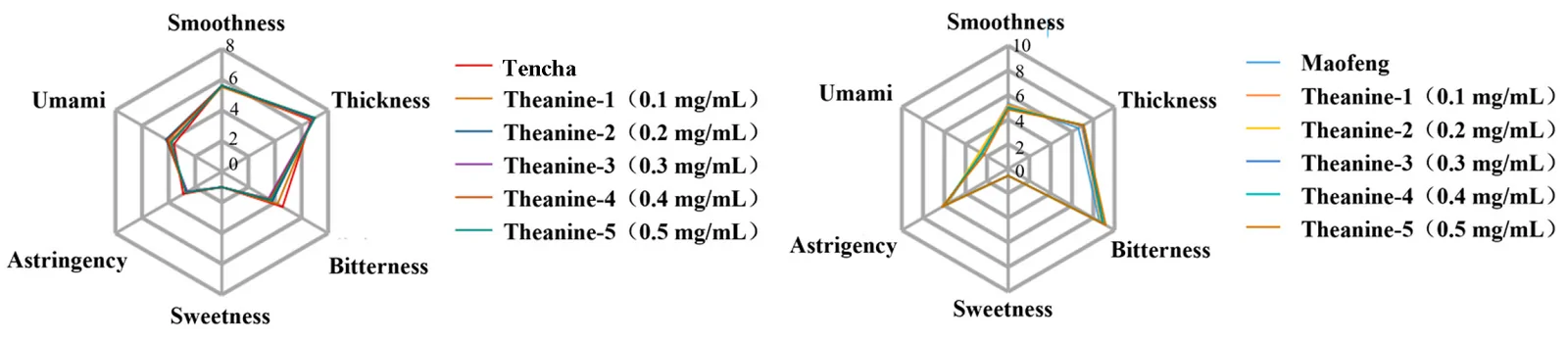
The effect of theanine addition on the infusion taste of Tencha and Maofeng.

Thea is the most abundant amino acid in tea, but its umami threshold of approximately 1.05 mg/mL generally higher than its concentration in tea infusions, suggesting a limited direct contribution to umami. In addition, Thea exhibits a mixed taste profile rather than a pure umami sensation at higher concentrations [39], which may explain why it was not identified by WGCNA as a key umami-associated compound. In our study, Thea showed distinct matrix-dependent effects in the two tea infusions (Figure 3). Thea may enhance umami through interactions with Glu and other taste-active compounds [40]. In Maofeng infusion, supplementation with 0.3 and 0.5 mg/mL Thea increased the bitterness score from 8.6 (control) to 8.9 and 9.2, respectively. By contrast, in Tencha infusion, 0.3 mg/mL Thea significantly decreased the bitterness score from 4.6 to 3.5, while a further increase to 0.5 mg/mL slightly raised the score to 3.7. This difference may be associated with the higher Glu level in Tencha infusion (125.45 μg/mL) than in Maofeng infusion (83.41 μg/mL), which may facilitate the interaction between Glu and Thea and contribute to bitterness suppression. Kaneko et al. reported that increasing the level of theanine enhanced the umami intensity of L-glutamate, suggesting that the sensory effect of theanine depends on its interaction with an umami-active matrix rather than on its intrinsic taste alone [41]. The higher Glu concentration in Tencha may therefore provide a more favorable background for the taste-modulating effect of added theanine.

### 3.4. Validation of the Taste-Modulating Effects of Asp

**Figure 4:**
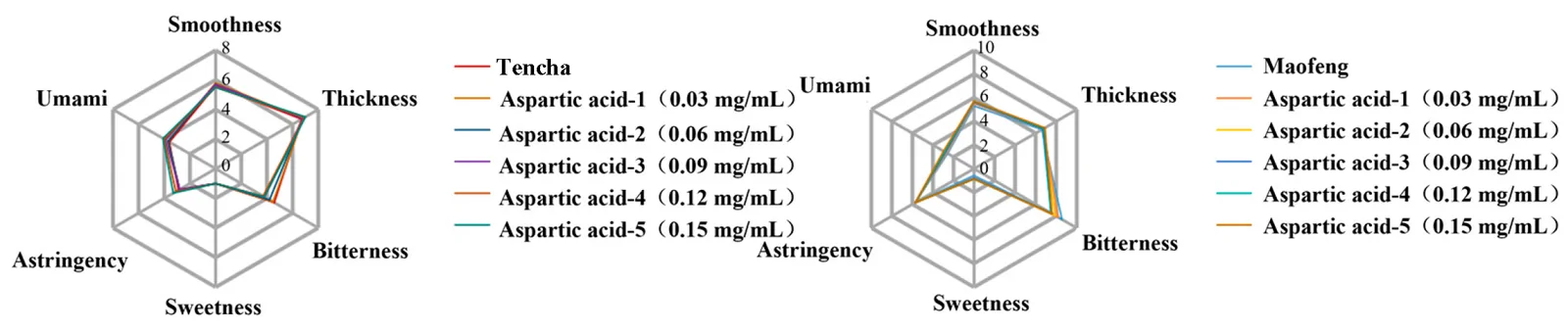
The effect of aspartic acid addition on the infusion taste of Tencha and Maofeng.

Figure 4 illustrates the effect of Asp addition on the sensory attributes of Maofeng and Tencha infusions. Asp decreased bitterness in both tea matrices, and the maximum reduction was observed at 0.12 mg/mL. At this concentration, the bitterness scores decreased from 8.6 to 7.5 in Maofeng and from 4.6 to 3.7 in Tencha. However, further increasing Asp to 0.15 mg/mL resulted in a weakened bitterness-reducing effect, although the scores remained below the control levels. These results suggest that Asp-mediated bitterness modulation was concentration-dependent and exhibited an optimal effective concentration range. Asp is an umami-active amino acid with a low sensory threshold of approximately 0.02 mg/mL, which may contribute to its bitterness-masking effect [42]. Asp (0.13–0.50 mg/mL) significantly reduced the bitterness of EGCG and ECG, whereas it enhanced the bitterness of GCG at high GCG concentrations, although this enhancement weakened as Asp concentration increased [16]. Tencha contained substantially more Asp but markedly less EGCG than Maofeng, which may partly explain the different changes in bitterness observed after Asp spiking.

### 3.5. Validation of the Taste-Modulating Effects of Arg

**Figure 5:**
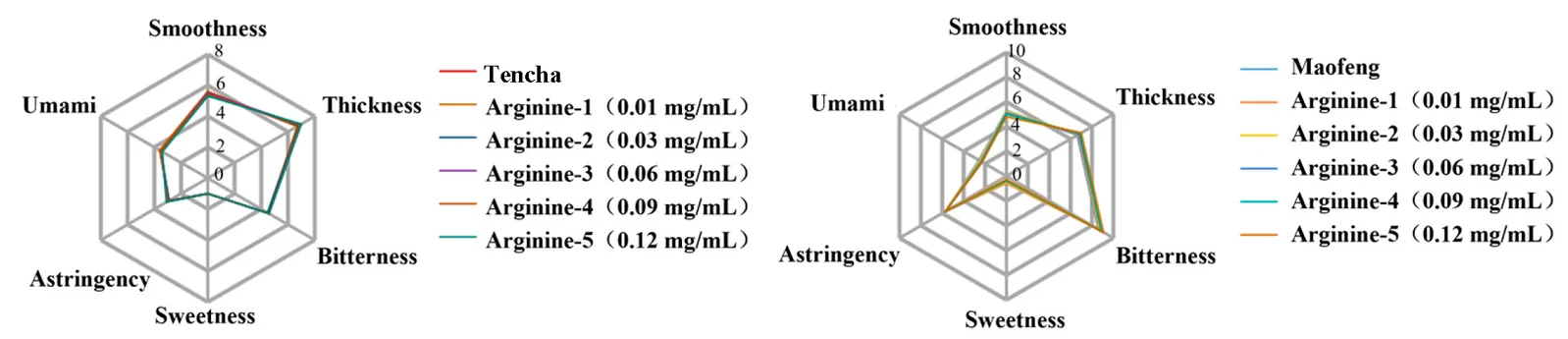
The effect of arginine addition on the infusion taste of Tencha and Maofeng.

Figure 5 shows the effects of Arg supplementation on the sensory evaluation scores of Maofeng and Tencha infusions. Arg exerted a limited effect on bitterness, particularly in Tencha. At the concentration of 0.01 and 0.03 mg/mL, Arg slightly increased the bitterness scores of Maofeng to 8.7 and 8.8, while the score of Tencha remained unchanged at 4.5. Even at 0.12 mg/mL, the bitterness scores increased only modestly to 9.1 in Maofeng and 4.6 in Tencha. These small changes indicate that Arg contributed little to bitterness under the tested conditions. This may be attributed to its relatively high bitterness threshold.

The matrix-dependent effects observed in this study indicate that bitterness and astringency modulation in jasmine tea is influenced by the compositional background of the tea base, particularly the relative levels of catechins and free amino acids. In complex tea infusions, exogenous sweet- and umami-active compounds may interact with endogenous taste constituents, resulting in compound-dependent and matrix-specific sensory responses. These findings suggest that the regulation of jasmine tea taste is governed by both the intrinsic chemical composition of the tea base and inter-component interactions within the infusion matrix.

## 4. Conclusions

This study combined WGCNA and spiking experiments to identify potential taste-active compounds involved in bitterness and astringency modulation in jasmine green tea. C, GCG, Asp, Glu, Arg, and Ala were associated with higher sweetness and umami and lower bitterness and astringency, while the spiking experiments confirmed that these effects were concentration- and matrix-dependent. Overall, jasmine tea taste appears to be regulated by the combined effects of multiple compounds rather than by individual constituents alone. These findings may provide a basis for tea-base selection and taste optimization. However, the spiking experiments were performed using only two representative tea matrices and a limited sensory panel size (six assessors), which may somehow limit the generalizability of the findings across different tea bases. Further investigations involving a broader selection of tea matrices and targeted mechanistic studies are needed to confirm the applicability of these taste-modulating effects and clarify the molecular interactions underlying bitterness and astringency regulation.

## Figures and Tables

**Table 1:** The concentrations of major flavor compounds in the infusions of four tea bases and the corresponding jasmine teas.

Compounds	Curly-Strip Maofeng			Orchid-Shaped Type Maofeng			Flat-Shaped Tea			Tencha		
(µg/mL)	Tea Base	S30	S35	TEA BASE	S30	S35	Tea Base	S30	S35	Tea Base	S30	S35
Catechin compounds												
GC	10.21 ± 1.88a	10.55 ± 0.84a	11.56 ± 1.11a	10.28 ± 0.55a	9.71 ± 0.49a	10.57 ± 0.81a	16.16 ± 1.47a	14.91 ± 0.60a	14.97 ± 1.45a	11.67 ± 0.80a	11.97 ± 0.53a	12.31 ± 0.89a
EGC	92.65 ± 1.72ab	94.05 ± 3.16ab	105.71 ± 14.87a	83.40 ± 1.55ab	91.98 ± 4.91a	78.31 ± 7.27b	97.72 ± 7.39a	98.79 ± 3.73a	100.46 ± 7.39a	126.80 ± 2.62a	124.45 ± 1.08a	124.36 ± 4.70a
C	13.30 ± 0.29a	13.34 ± 0.52a	12.78 ± 1.37a	14.35 ± 0.62a	14.52 ± 0.96a	13.88 ± 0.35a	13.77 ± 1.01a	13.70 ± 1.14a	15.08 ± 1.21a	7.23 ± 0.37a	7.16 ± 0.50a	6.53 ± 0.84a
EC	33.00 ± 2.69a	31.49 ± 2.06ab	34.24 ± 4.87a	27.96 ± 1.08a	28.69 ± 1.65a	29.20 ± 1.18a	33.42 ± 2.49a	35.51 ± 1.79a	34.24 ± 1.95a	36.28 ± 0.46a	37.14 ± 0.59a	36.89 ± 0.74a
EGCG	39.11 ± 3.25a	35.93 ± 3.37ab	35.19 ± 1.37ab	46.63 ± 3.43a	46.10 ± 1.99a	45.49 ± 1.28a	89.61 ± 0.99a	89.72 ± 2.29a	88.26 ± 2.61a	17.44 ± 1.47a	17.44 ± 1.32a	16.76 ± 1.52ab
GCG	9.70 ± 1.11a	10.19 ± 0.14a	10.40 ± 0.53a	10.54 ± 0.16a	9.71 ± 0.28a	10.46 ± 0.23a	11.33 ± 0.86a	10.58 ± 0.78a	11.71 ± 0.87a	4.19 ± 0.58a	4.05 ± 0.18a	4.33 ± 0.36a
ECG	10.08 ± 0.60a	9.75 ± 0.68a	9.15 ± 0.24a	9.94 ± 0.55a	9.77 ± 0.63a	10.24 ± 0.20a	13.42 ± 0.99a	13.52 ± 0.93a	12.83 ± 1.20a	8.23 ± 1.32ab	9.32 ± 0.92a	9.06 ± 0.33a
CG	1.56 ± 0.12a	1.63 ± 0.09a	1.51 ± 0.08a	1.45 ± 0.09a	1.57 ± 0.07a	1.60 ± 0.11a	3.12 ± 0.26a	3.00 ± 0.13a	3.04 ± 0.14a	1.25 ± 0.05a	1.15 ± 0.09a	1.20 ± 0.06a
TC	209.62 ± 1.42a	206.94 ± 2.12a	220.55 ± 16.52a	204.55 ± 3.83a	212.03 ± 5.17a	199.75 ± 7.93a	278.53 ± 7.26a	279.75 ± 6.09a	280.59 ± 8.42a	213.09 ± 0.76a	212.68 ± 0.73a	211.44 ± 3.82a
Flavonol glycosides												
M-gal-rha-glu	0.57 ± 0.01a	0.56 ± 0.06a	0.53 ± 0.07a	0.50 ± 0.04ab	0.52 ± 0.01a	0.52 ± 0.04a	0.65 ± 0.04a	0.64 ± 0.02a	0.66 ± 0.03a	0.63 ± 0.12a	0.59 ± 0.08ab	0.48 ± 0.05ab
M-gal	0.37 ± 0.03a	0.34 ± 0.02a	0.33 ± 0.04a	0.31 ± 0.03a	0.32 ± 0.03a	0.30 ± 0.02a	0.40 ± 0.02a	0.44 ± 0.03a	0.42 ± 0.01a	0.49 ± 0.03a	0.48 ± 0.04a	0.47 ± 0.03a
M-glu	0.48 ± 0.09a	0.46 ± 0.11ab	0.46 ± 0.13ab	0.39 ± 0.05a	0.41 ± 0.04a	0.40 ± 0.02a	0.50 ± 0.01a	0.52 ± 0.02a	0.51 ± 0.02a	0.61 ± 0.06a	0.59 ± 0.05a	0.62 ± 0.07a
Q-gal-rha-glu	3.37 ± 0.10a	3.45 ± 0.20a	3.43 ± 0.21a	3.24 ± 0.25a	3.19 ± 0.28a	3.32 ± 0.30a	3.40 ± 0.21a	3.53 ± 0.17a	3.51 ± 0.29a	4.45 ± 0.16a	4.38 ± 0.38a	4.55 ± 0.12a
Q-glu-rha-glu	5.14 ± 0.23a	5.47 ± 0.47a	5.55 ± 0.33a	3.28 ± 0.20a	3.44 ± 0.19a	3.41 ± 0.35a	5.86 ± 0.29a	5.81 ± 0.27a	5.74 ± 0.33a	5.98 ± 0.11a	6.06 ± 0.24a	5.82 ± 0.25a
Q-glu-rha-rha	7.24 ± 0.08c	8.14 ± 0.11a	8.15 ± 0.12a	6.58 ± 0.07b	8.53 ± 0.61a	8.38 ± 0.53a	5.94 ± 0.19b	7.41 ± 0.17a	7.56 ± 0.33a	10.12 ± 0.42b	13.24 ± 0.64a	12.96 ± 1.21a
Q-gal	0.85 ± 0.14a	0.94 ± 0.05a	0.95 ± 0.01a	0.81 ± 0.07a	0.84 ± 0.04a	0.82 ± 0.05a	0.85 ± 0.01a	0.88 ± 0.06a	0.88 ± 0.04a	0.99 ± 0.13a	0.90 ± 0.05a	0.80 ± 0.14a
Q-glu	0.20 ± 0.04a	0.24 ± 0.02a	0.24 ± 0.02a	0.12 ± 0.01a	0.12 ± 0.00a	0.12 ± 0.01a	0.32 ± 0.02a	0.33 ± 0.02a	0.34 ± 0.03a	0.14 ± 0.01a	0.14 ± 0.01a	0.13 ± 0.01a
K-glu-rha-glu	10.60 ± 0.28a	10.86 ± 0.74a	11.19 ± 0.48a	7.06 ± 0.57a	7.08 ± 0.63a	7.03 ± 0.23a	11.16 ± 0.86a	11.45 ± 0.72a	11.35 ± 0.45a	10.78 ± 0.31a	11.08 ± 0.99a	11.08 ± 0.32a
K-gal	1.55 ± 0.23a	1.54 ± 0.09a	1.69 ± 0.23a	1.44 ± 0.15a	1.49 ± 0.11a	1.47 ± 0.13a	0.76 ± 0.05a	0.78 ± 0.07a	0.78 ± 0.03a	0.73 ± 0.11a	0.77 ± 0.02a	0.73 ± 0.08a
K-glu-rha	1.92 ± 0.19a	2.08 ± 0.11a	2.11 ± 0.17a	1.95 ± 0.17a	1.99 ± 0.07a	2.01 ± 0.03a	1.43 ± 0.14a	1.47 ± 0.08a	1.46 ± 0.04a	0.90 ± 0.15a	0.79 ± 0.07a	0.84 ± 0.11a
K-glu	0.10 ± 0.01a	0.11 ± 0.01a	0.10 ± 0.01a	0.11 ± 0.01a	0.11 ± 0.01a	0.11 ± 0.01a	0.15 ± 0.01a	0.15 ± 0.02a	0.15 ± 0.00a	0.10 ± 0.01a	0.10 ± 0.01a	0.09 ± 0.02a
TFG	32.41 ± 0.61b	34.19 ± 1.02a	34.75 ± 0.72a	25.78 ± 0.24b	28.04 ± 0.22a	27.90 ± 0.45a	31.42 ± 0.75b	33.40 ± 0.68a	33.35 ± 0.82a	35.93 ± 1.33b	39.12 ± 0.43a	38.57 ± 0.38a
Amino acids												
Asp	47.29 ± 2.15a	39.81 ± 2.37b	44.96 ± 1.99ab	46.78 ± 3.83a	41.01 ± 2.98a	33.72 ± 1.11b	55.81 ± 0.91a	42.54 ± 1.60b	45.21 ± 1.82b	110.53 ± 2.62a	101.60 ± 1.32b	95.82 ± 1.41c
Glu	83.41 ± 4.03a	66.89 ± 4.02b	68.95 ± 3.56b	64.12 ± 2.26a	57.88 ± 3.44a	46.82 ± 2.79b	60.13 ± 1.55a	42.52 ± 1.39b	40.76 ± 1.44b	125.45 ± 9.13a	116.73 ± 1.65ab	106.40 ± 1.06b
Asn	13.92 ± 0.76a	13.66 ± 0.66a	12.96 ± 0.80a	31.25 ± 2.63a	22.21 ± 1.38b	21.70 ± 0.61b	46.28 ± 0.81ab	46.22 ± 2.11a	29.96 ± 0.76b	36.64 ± 0.80a	24.90 ± 0.34d	34.34 ± 0.31b
Ser	22.03 ± 0.48a	20.58 ± 2.04a	20.98 ± 1.91a	20.71 ± 1.62a	13.64 ± 0.19bc	11.69 ± 0.95c	17.08 ± 1.11a	20.01 ± 2.06a	16.87 ± 1.65ab	26.60 ± 1.61a	20.98 ± 0.54b	20.60 ± 0.08b
Gln	77.12 ± 3.88a	80.00 ± 7.46a	51.13 ± 2.14b	42.40 ± 1.69a	44.18 ± 0.93a	28.29 ± 0.70b	71.82 ± 1.44a	53.58 ± 1.42b	55.86 ± 2.41b	42.03 ± 0.62a	26.62 ± 0.32c	28.42 ± 0.18c
His	3.78 ± 0.47a	4.24 ± 1.02a	4.40 ± 0.41a	5.86 ± 0.37a	4.32 ± 0.29b	3.74 ± 0.28b	7.28 ± 0.64a	6.00 ± 0.33b	6.09 ± 0.57b	7.40 ± 0.65a	5.99 ± 0.14b	6.29 ± 0.51b
Gly	4.62 ± 0.41a	5.68 ± 0.54a	4.69 ± 0.42a	8.33 ± 0.75a	3.96 ± 0.35c	4.19 ± 0.38c	6.92 ± 0.69a	6.92 ± 0.62a	5.20 ± 0.42b	4.35 ± 0.17a	2.58 ± 0.18c	3.18 ± 0.22b
Thr	9.41 ± 0.41a	8.01 ± 0.23b	8.30 ± 0.11b	10.15 ± 0.74a	7.16 ± 0.14b	6.30 ± 0.43b	10.06 ± 0.54a	9.07 ± 0.42ab	8.28 ± 0.74b	13.24 ± 0.19a	9.16 ± 0.10c	9.86 ± 0.10b
Arg	24.80 ± 2.36ab	28.76 ± 1.69a	24.87 ± 1.06ab	23.86 ± 2.04a	21.81 ± 2.08a	12.16 ± 0.43c	31.03 ± 2.02a	33.89 ± 1.27a	22.15 ± 0.90b	86.68 ± 4.14a	81.06 ± 4.99a	70.72 ± 0.93b
Ala	10.46 ± 0.63a	9.94 ± 0.84a	11.22 ± 0.33a	9.83 ± 0.95a	8.01 ± 0.77b	8.44 ± 0.69ab	8.53 ± 0.50a	8.50 ± 0.82a	8.30 ± 0.78a	24.01 ± 0.33a	17.54 ± 0.21c	18.47 ± 0.05b
GABA	8.75 ± 0.29a	7.11 ± 0.29bc	6.51 ± 0.51c	4.60 ± 0.41a	3.03 ± 0.07b	3.63 ± 0.14b	4.04 ± 0.26a	3.46 ± 0.13b	3.26 ± 0.07b	6.53 ± 0.53a	4.12 ± 0.07b	4.02 ± 0.17b
Thea	378.68 ± 17.70a	271.56 ± 15.98b	298.90 ± 10.26b	274.13 ± 12.89ab	276.88 ± 24.51a	231.20 ± 5.52b	238.24 ± 7.64ab	208.76 ± 7.77b	229.48 ± 2.82a	358.21 ± 9.45a	271.34 ± 3.92b	237.64 ± 6.37c
Tyr	7.21 ± 0.32a	4.08 ± 0.21c	5.31 ± 0.52b	9.67 ± 0.41a	4.55 ± 0.40c	3.10 ± 0.21d	7.63 ± 0.72a	7.88 ± 0.11a	7.48 ± 0.51a	7.89 ± 0.19a	4.62 ± 0.05d	4.99 ± 0.09c
Val	4.72 ± 0.13ab	3.99 ± 0.26c	5.23 ± 0.35a	10.41 ± 0.89a	6.60 ± 0.55b	5.17 ± 0.36b	11.24 ± 0.90a	11.04 ± 0.16a	9.09 ± 0.50b	6.70 ± 0.35a	4.38 ± 0.05c	5.83 ± 0.50b
Met	2.69 ± 0.09a	2.10 ± 0.10b	1.47 ± 0.12c	1.92 ± 0.36a	1.99 ± 0.20a	0.09 ± 0.01c	1.58 ± 0.60a	0.96 ± 0.04a	1.09 ± 0.15a	1.43 ± 0.37a	1.07 ± 0.06a	1.42 ± 0.19a
Trp	7.35 ± 0.25a	4.15 ± 0.34bc	3.75 ± 0.30c	9.23 ± 0.50a	3.84 ± 0.36b	4.46 ± 0.42b	9.40 ± 0.63a	6.57 ± 0.50c	7.62 ± 0.35b	9.03 ± 0.62a	6.19 ± 0.05bc	7.26 ± 0.52b
Phe	10.12 ± 0.38a	6.69 ± 0.06b	9.17 ± 1.57a	12.66 ± 1.02a	9.87 ± 0.72b	10.46 ± 0.81b	14.16 ± 1.40a	13.57 ± 0.57a	13.04 ± 0.53a	10.60 ± 0.80a	6.94 ± 0.12c	8.80 ± 0.62b
Ile	5.83 ± 0.11a	3.86 ± 0.24c	4.57 ± 0.42b	8.46 ± 0.46a	4.69 ± 0.29b	4.64 ± 0.41b	8.87 ± 0.66a	8.13 ± 0.49ab	7.36 ± 0.54b	8.34 ± 0.12a	6.35 ± 0.04c	6.75 ± 0.19b
Leu	9.19 ± 0.32a	7.53 ± 0.25b	7.33 ± 0.66b	13.42 ± 0.96a	6.60 ± 0.07b	6.18 ± 0.48b	10.57 ± 0.97a	9.69 ± 0.26ab	8.55 ± 0.75b	8.08 ± 0.80a	5.70 ± 0.34b	6.34 ± 0.55b
Lys	9.25 ± 0.68a	7.23 ± 0.45b	7.67 ± 0.49b	13.38 ± 0.89a	8.43 ± 1.60b	8.12 ± 0.75b	10.09 ± 0.68a	9.85 ± 0.82a	9.15 ± 0.83a	10.54 ± 0.93a	7.91 ± 0.02b	8.08 ± 0.71b
TAA	740.61 ± 29.68a	595.88 ± 15.72b	602.38 ± 6.71b	621.17 ± 24.76a	550.66 ± 33.27b	454.09 ± 15.19c	630.77 ± 16.72a	549.16 ± 14.98b	534.79 ± 17.75b	904.26 ± 9.02a	725.78 ± 11.01b	685.22 ± 5.41c
Caffeine	213.84 ± 3.77a	205.42 ± 14.32a	208.73 ± 9.56a	211.46 ± 6.84a	212.90 ± 7.46a	201.14 ± 18.34a	217.39 ± 1.53a	214.74 ± 9.43a	207.43 ± 6.46a	223.92 ± 1.14a	224.76 ± 16.85a	223.24 ± 2.37a

Note: GC, gallocatechin; EGC, epigallocatechin; C, catechin; EC, epicatechin; EGCG, epigallocatechin gallate; GCG, gallocatechin gallate; ECG, epicatechin gallate; CG, catechin gallate; TC, total catechins; Asp, aspartic acid; Glu, glutamic acid; Asn, asparagine; Ser, serine; Gln, glutamine; His, histidine; Gly, glycine; Thr, threonine; Arg, arginine; Ala, alanine; GABA, γ-aminobutyric acid; Thea, theanine; Tyr, tyrosine; Val, valine; Met, methionine; Trp, tryptophan; Phe, phenylalanine; Ile, isoleucine; Leu, leucine; Lys, lysine; TAA, total amino acids; M-gal-rha-glu, myricetin-3-*O*-galactosyl-rhamnosyl-glucoside; M-gal, myricetin-3-*O*-galactoside; M-glu, myricetin-3-*O*-glucoside; Q-gal-rha-glu, quercetin-3-*O*-galactosyl-rhamnosyl-glucoside; Q-glu-rha-glu, quercetin-3-*O*-glucosyl-rhamnosyl-glucoside; Q-glu-rha-rha, quercetin-3-*O*-rhamnosyl-rhamnosyl-glucoside; Q-glu, quercetin-3-*O*-glucoside; Q-gal, quercetin-3-*O*-galactoside; K-glu-rha-glu, kaempferol-3-*O*-glucosyl-rhamnosyl-glucoside; K-glu, kaempferol-3-*O*-glucoside; K-gal, kaempferol-3-*O*-galactoside; K-glu-rha, kaempferol-3-*O*-rhamnosyl-glucoside; TFG, total flavonol glycosides. Data are expressed as the mean ± SD. Within the same row, different letters indicate significant differences among the differently treated samples from the same tea base (*p* < 0.05).

## Data Availability

The original contributions presented in this study are included in the article. Further inquiries can be directed to the corresponding authors.

## Abbreviations

The following abbreviations are used in this manuscript:

WGCNA	Weighted gene co-expression network analysis
EGCG	Epigallocatechin gallate
EGC	Epigallocatechin
ECG	Epicatechin gallate
EC	Epicatechin
GCG	Gallocatechin gallate
GC	Gallocatechin
CG	Catechin gallate
C	Catechin
M-gal-rha-glu	Myricetin-3-*O*-galactosyl-rhamnosyl-glucoside
M-gal	Myricetin 3-*O*-galactoside
M-glu	Myricetin 3-*O*-glucoside
Q-gal-rha-glu	Quercetin-3-*O*-glucosyl-rhamnosyl-galactoside
Q-glu-rha-glu	Quercetin-3-*O*-glucosyl-rhamnosyl-glucoside
Q-glu-rha-rha	Quercetin-3-*O*-rhamnosyl-rhamnosyl-glucoside
Q-gal	Quercetin 3-*O*-galactoside
Q-glu	Quercetin 3-*O*-glucoside
K-glu-rha-glu	Kaempferol-3-*O*-glucosyl-rhamnosyl-glucoside
K-gal	Kaempferol-3-*O*-galactoside
K-glu-rha	Kaempferol-3-*O*-rhamnosyl-glucoside
K-glu	Kaempferol 3-*O*-glucoside
Asp	Aspartic acid
Glu	Glutamic acid
Asn	Asparagine
Ser	Serine
Gln	Glutamine
His	Histidine
Gly	Glycine
Thr	Threonine
Arg	Arginine
Ala	Alanine
GABA	γ-aminobutyric acid
Thea	Theanine
Tyr	Tyrosine
Val	Valine
Met	Methionine
Trp	Tryptophan
Phe	Phenylalanine
Ile	Isoleucine
Leu	Leucine
Lys	Lysine
OPA	O-phthalaldehyde
QDA	Quantitative descriptive analysis
TC	Total catechins
TFG	Total flavonol glycosides
TAA	Total amino acids
